# Aspects of the lifestyle of multipartite viruses apply to monopartite segmented and perhaps nonsegmented viruses

**DOI:** 10.1038/s44298-024-00045-1

**Published:** 2024-07-25

**Authors:** Yannis Michalakis, Stéphane Blanc

**Affiliations:** 1 MIVEGEC, Université Montpellier, CNRS, IRD, Montpellier, France; 2 PHIM, Université Montpellier, IRD, CIRAD, INRAE, Institut Agro, Montpellier, France

**Keywords:** Viral evolution, Virus-host interactions

## Abstract

Recent research on faba bean necrotic stunt virus, aiming to understand how multipartite viruses function and potentially their existence, revealed three surprising features: a non-uniform segment frequency distribution (genome formula), a multicellular functioning, and the non-concomitant transmission of genomic segments. We review the occurrence of these features in other multipartite viruses and discuss their potential operation in monopartite viruses with segmented genomes and perhaps even in viruses with nonsegmented genomes.

## Introduction

The existence of multipartite viruses, i.e. viruses with a genome consisting of several segments, each packaged in a different viral particle, is intriguing because they must find a way to keep their genomic information together during within-host colonization and between-host transmission. In addition, no clear general advantage specific to this genomic architecture has yet been identified^1,2^. A series of studies on faba bean necrotic stunt virus (FBNSV; family *Nanoviridae*, genus *Nanovirus*) aiming to understand how multipartite viruses function and potentially their existence revealed three surprising features: a genome formula, a multicellular lifestyle, and the possibility of non-concomitant transmission of genomic segments.

The genome of FBNSV consists of eight separately encapsidated circular single-stranded (ss) DNA segments, each of about 1 kb and encoding a single gene. The different segments of FBNSV do not accumulate at equal frequencies within hosts^3^. Instead, they reproducibly accumulate at a frequency distribution where some segments largely dominate while others are rare in an active infection^3^. This frequency distribution, termed genome formula (GF), is host-specific^3^, impacts gene expression within hosts^4^, and differs from that in insect vectors^5^. The fact that the GF changes ‘immediately’ upon host species switch suggests that it may represent a mechanism allowing rapid adaptation of the virus to variation in its environment. This notion was theoretically investigated and confirmed^6^, even though the adaptive nature of the genome formula has yet to be experimentally confirmed (but see further discussion below). The non-uniform frequency distribution nevertheless adds to the issue of genome integrity because it is a priori easier to lose rare segments.

FBNSV can produce functional infections even when its genomic segments do not cooccur within individual host cells; in fact, more often than not they do not. Instead, gene products traffic between host cells such that proteins produced by one segment often accumulate in cells containing other segments but where their cognate segment is missing. This viral system is thus complemented and functional at a multicellular scale^7^.

Lastly, the different segments of FBNSV do not need to be transmitted together but can be acquired and inoculated separately^8^. This can occur when different insect vector individuals acquire distinct segment subsets from different host individuals and together inoculate them in the same host. The complete genome can then be reconstituted in the recipient host. This can also occur when the same vector individual sequentially acquires distinct segment subsets from different hosts and inoculates them all at once in the recipient host. In this latter case, the complete genome is reconstituted in the vector.

Thus, the maintenance of genome integrity is challenged in FBNSV by the non-uniform GF, but this challenge is mitigated by the fact that genome integrity is not mandatory either at the individual cell level or during between-host transmission. Below, we rapidly review evidence on the generality of these intriguing processes*,* i.e. whether they occur in other multipartite viruses, in the monopartite segmented viruses, which package their genomic segments together within each viral particle, or even in monopartite nonsegmented viruses^9^.

## Multipartite viruses

Among these processes, the one that has been most extensively investigated so far, perhaps because of its earlier discovery, is the GF (Table 1). Several studies on multipartite viruses, although still limited in number, cover a rather diverse set of viruses with respect to the type of nucleic acid bearing the genetic information (ssDNA, (+)ssRNA, and (−)ssRNA). It should be noted, however, that most of these studies bear on ssDNA viruses while the majority of multipartite viruses are RNA viruses^1,10^.

**Table 1:** Viruses for which the existence of a genome formula and its potential variation were investigated

Genomic architecture	Nucleic acid	Family	H	Species	S	GF	HT	Or	Vc	Other	Eq
Multipartite	ssDNA	*Nanoviridae*	p	FBNSV	8	!=^3^	+^3^		+^5^		ft^3^
			p	FBNYV	8	!=^52^			+^52^	sat^52^	
			p	BBTV	6	!=^19–21^		^a^^19^	+^20^	sat^20,21^	
		*Geminiviridae*	p	ACMV	2	=^12^			+^12^	co^12^	
			p	EACMV	2	=^12^			+^12^	co^12^	
			p	SLCCNV	2	!=^11^	+^11^				ft^11^
			p	SLCMV	2	=^11^					t^11^
		*Bidensoviridae*	i	BmBDV	2	!=^13^		+^13^	NA		
	ssRNA (+)	*Bromoviridae*	p	AMV	3	!=^14,15^	+^15^				ft^15^
			p	CMV	3	!=^17,53^	−^17^			sat^53^ co^18^ ^b^	
		*Benyviridae*	p	BNYVV	5	!=^54^	+^54^	+^54^			f^54^
	ssRNA (−)	*Phenuiviridae*	pi	RSV	4	!=^39^			+^39^		
Monopartite segmented	ssRNA (−)	*Phenuiviridae*	mi	RVFV	3	!=^37,42^			+^42^		
		*Tospoviridae*	pi	TSWV	3	!=^40^					
		*Orthomyxoviridae*	v	IAV	8	!=^31^					
	dsRNA	*Reoviridae*	mi	BTV	10	!=^36^			+^36^		

H: host, plant (p), insect (i), vertebrate (v) or mammal (m); (pi) refers to viruses that have been shown to replicate both in the plant host and the insect vector and (mi) to viruses which have been shown to replicate both in the mammal host and the insect vector. This is still debated for begomoviruses (all *Geminiviridae* in this table) which we classified as (p) for simplicity; S: number of genomic segments; GF: (=) even distribution of segment relative frequencies or (!=) otherwise; HT: whether GF differs across host species (+) or not (−); Or: whether GF differs across organs (+) or not (−); Vc: whether GF differs between host and vector (+) or not (−); Other: effects of other factors, i.e. presence of virus satellites (sat) or coinfection with other viruses (co); Eq: authors investigated whether the GF corresponded to equilibrium (i.e. ‘setpoint GF’) either by testing whether infections converge to the same GF when starting from different initial frequencies of segments (f), or by following GF over time during the course of an infection (t) or both (ft). Numbers indicate references. Blank cells in these columns indicate the question has not been investigated. NA indicates the question is not relevant, e.g. Vc column for non-vector transmitted viruses.

^a^In this paper, the authors report concentrations of genomic segments in different host organs but do not test whether these differ statistically.

^b^In this study, the authors firmly conclude on an effect of coinfection on the variance of GF but not on the GF itself.

A first trend that appears from these studies is that with few exceptions, the GF corresponds to a non-uniform distribution of segment frequencies: as in FBNSV, some segments are rare, and others are frequent. The few cases of equifrequency have so far been observed exclusively in the genus *Begomovirus*^11,12^ (family *Geminiviridae*), some members of which are bipartite ssDNA viruses with each of their two segments encoding more than one gene. This observed equifrequency cannot be considered a characteristic of this genus since one begomovirus with a 5–6 ratio of its segments’ frequency has been identified^11^ while two other begomoviruses with equifrequency in the plant host show different segment ratios in their vector^12^. Moreover, equifrequency cannot be viewed as a feature of bipartite viruses either since the bipartite ssDNA bidensoviruses (family *Bidnaviridae*, genus *Bidnavirus*) exhibit a GF with unequal segment frequencies^13^. Finally, equifrequency cannot be explained by the fact that the distinct segments encode several genes, as the bidensoviruses^13^ and tripartite ssRNA positive sense (+) bromoviruses^14–17^ (family *Bromoviridae*, genus *Bromovirus*) also have segments which carry more than one ORF and a GF with unequal segment frequencies.

As mentioned above, GF was hypothesized to represent a mechanism enabling multipartite viruses to rapidly adjust their gene expression to environmental heterogeneity through gene copy number variation^3,6^. In agreement with this hypothesis, almost all studies asking whether GF varies across ‘environments’ found that it does. This is the case whether the ‘environment’ corresponds to different hosts, a passage from host to insect vector, different organs within an individual host, or coinfection with other genomic entities such as satellites (Table 1). The only exception is ssRNA(+) tripartite cucumber mosaic virus (CMV), a bromovirus, the mean GF of which does not seem to change across host plant species^17^ nor in the presence of coinfecting viruses^18^, though in the latter case, coinfection significantly decreases the GF variance. The latter study interestingly discusses that this lack of effect could be due to the quantification technique used. Indeed, in ssRNA(+) viruses, genomic and messenger RNAs are sometimes co-detected and it can thus be tricky (when possible at all) to distinguish gene copy number from gene expression variation. This is relevant because, as these authors note and further explained in the next paragraph, gene expression was shown to be much more similar across host species than GF in FBNSV^4^, the only virus where this comparison was explicitly performed.

The study by Gallet et al.^4^ showed that the GF indeed has functional implications as gene copy number variation correlates with mRNA variation in hosts of two species: plants of each species with a higher copy number of a gene also have higher titers of the corresponding mRNA, indicating that GF indeed affects gene expression^4^. Perhaps counterintuitively, the GF differed much more between hosts than the corresponding formulae based on mRNA distributions. This arose because the slopes of the mRNA-to-DNA relationships differed across segments and for some segments across hosts. These differing slopes indicate different mRNA/gene copy ratios, something also observed in other studies on ssDNA viruses^11,19–21^. Therefore, gene expression regulation may arise from processes occurring at two levels. One is acting at the individual segment level and is linked to segment-specific regulatory sequences. For example, the promotor strength or mRNA stability can depend on the segment^4,21^, the host species^4^, or even the organ^19^, leading to different mRNA/gene copy ratios. The second level is acting at the population of segments level through the observed gene copy number variation resulting in the differing GF. These two regulation levels must not be confused and are complementary.

The adaptive nature of the GF, although intuitively and theoretically appealing, remains yet to be demonstrated. No study has unequivocally shown that GF variation affects even a fitness component. In FBNSV, virus titer increases as the infection converges to setpoint GF, i.e. the GF at equilibrium, but this relationship was not statistically confirmed^3^. The only other virus where the question was investigated is a bromovirus, ssRNA (+) alfalfa mosaic virus (AMV)^15^. It was shown that total RNA titer but not encapsidated RNA was maximized at the setpoint GF pointing at a possible increased expression but no detectable higher viral accumulation.

These and many other questions beg for further investigation. For example, how does the GF arise? A recent study showed that processes at the individual segment and at the group of segment levels concur to establish the GF of FBNSV in a host plant^22^, but a lot remains to be elucidated on the mechanisms leading to the GF. It would also be important to study how the GF interacts with host physiology and resistance/tolerance mechanisms. Finally, it would be worth investigating whether there is polymorphism within viruses for their GF reaction norms which would suggest, assuming such polymorphism has a genetic basis, that GF can evolve.

## Monopartite segmented viruses

It is intuitive to think that monopartite segmented viruses, because they package their segments in the same virion and because some of them are known to have specific packaging mechanisms, should package one copy of each segment in each viral particle and thus have equal frequencies for all their segments within hosts. These intuitions, however, proved wrong, at least in a number of cases and on several accounts. First, the fact that a virus possesses specific packaging mechanisms does not preclude the production of viral particles that do not contain all genomic segments. This is the case, for example, for the influenza A virus (IAV; family *Orthomyxoviridae*, genus *Alphainfluenzavirus*), which is known to package its eight genomic segments using specific packaging mechanisms in a “7 + 1” configuration^23,24^. Yet, this virus has been shown to produce very large proportions of ‘semi-infectious particles’^25^ or SIPs, which are particles unable to express at least one viral protein (for a review, see Diefenbacher and colleagues^26^). Several mechanisms could give rise to SIPs, and the lack of genomic segments has been demonstrated to be one of them^27^. A detailed investigation revealed that, on average, an IAV segment has a 58% probability of being replicated in a host cell when introduced by a single virion, this probability varying from 50% to 67% depending on the segment^28^. These probabilities, along with other evidence, imply that coinfection, allowing complementation within host cells, is common in IAV^28–30^. Such observations led the authors of some of the above-cited publications to hypothesize that SIP production could be a way to allow IAV to modulate its gene expression in a manner analogous to the GF. This hypothesis has been supported further by the observation that a mutation in the nucleoprotein (NP) segment of an IAV strain strongly decreased the packaging, genomic accumulation, and gene expression of the neuramidinase (NA) segment, thus modifying the IAV GF^31^. Intriguingly, presumably through complementation, while this mutation increases the production of SIPs, it also enhances viral load. This study provides another example where GF modification appears linked to increased viral fitness, though here, as well, the potential causal relation between GF modification and increased fitness remains to be demonstrated^31^. As a final note on IAV, we would like to remark that even though the very definition of SIPs implies the non-expression of specific genes in individual cells and that coinfections by complementing SIPs are very common within hosts, the hypothesis that IAV could potentially function in a multicellular way of life, at least in hosts where multiplicity of infection would be low, has not been experimentally evaluated and thus remains a possibility. Indeed, it was recently shown that IAV stimulates the formation of cell-connecting tunneling nanotubes which it can use for cell-to-cell transfer of genome segments^32–34^ and viral proteins^32,33^. Beyond the relevance of this cell-to-cell trafficking for immune evasion, it was shown that through this mechanism, complementation and reassortment do not necessitate coinfection at the individual host cell level and that genome segments can move across host cells in a virion-free way^34^. Thus, the features characterizing a multicellular way of life, i.e. the ability to complement viral functions across different host cells, are present in this monopartite segmented virus. What needs to be further characterized is the extent to which such complementation may involve exclusively trafficking of gene products and not that of genome components. Indeed, in the latter case, infections become cell-autonomous immediately after the transfer of the relevant genome segments.

Similarly to IAV, dsRNA orbiviruses in the order *Reovirales* also possess highly specific packaging mechanisms (reviewed by Borodavka and colleagues^35^). Nevertheless, a study on bluetongue virus (BTV) remarkably revealed not only that the 10 genomic segments accumulate at markedly different frequencies within hosts but, moreover, that they produce different GFs in hosts and vectors^36^.

It is perhaps less surprising that GFs were also found in viruses that are known for their less specific packaging such as members of the order *Bunyavirales*^37,38^. Indeed, a GF was found not only in multipartite rice stripe virus (RSV; family *Phenuiviridae*, genus *Tenuivirus*)^39^ but also in monopartite segmented tomato spotted wilt virus (TSWV; family *Tospoviridae*, genus *Orthotospovirus*)^40^ and Rift Valley fever virus (RVFV; family *Phenuiviridae*, genus *Phlebovirus*)^37,41^. The latter studies showed that bunyaviruses do not have a mechanism ensuring the incorporation of a complete set of segments in a single viral particle. They thus produce many viral particles, perhaps the majority, lacking genome segments. Most viruses of this order replicate in both hosts and vectors. A comparison between those that use plants as hosts and those that use animals could thus potentially help elucidate the still unresolved question of why multipartite viruses almost exclusively infect plants and fungi. Three examples illustrate this statement: (i) RVFV infects mammals and is transmitted by mosquitoes. It thus replicates solely in animals where it packages several segments together and clearly fits in the category of monopartite segmented viruses. Its packaging has been shown to be more efficient in insect (vector) cells than in mammalian (host) cells, resulting in a larger proportion of viral particles containing all genomic segments^42^. (ii) RSV infects plants and is transmitted by insect vectors. It does not form enveloped viral particles but propagates in both host plants and insect vectors in the form of three separate ribonucleoprotein filaments, each representing a genome segment. It fits in the category of multipartite viruses but, nevertheless, its GF appears more constant over time in the insect vector than in the host plant^39^. (iii) Finally, as a sort of intermediate stage, TSWV also infects plants and is transmitted by insects, but it does form enveloped viral particles. Interestingly, mutants lacking the viral envelope were nevertheless able to infect plants^43^, suggesting that the virus propagates within host plants as nucleoprotein filaments. A subsequent study showed that envelope-deficient mutants were unable to infect the vector midgut and vector primary cell cultures^44^, suggesting that the virus enters and propagates within insect vectors as fully assembled viral particles packaging several segments together. A definitive confirmation of such functioning would imply that TSWV adopts a multipartite lifestyle within the plant and switches to a monopartite segmented lifestyle within the insect vector. Further investigation of multipartite and monopartite segmented bunyaviruses transmitted by insects and infecting plants could reveal steps in the viral life cycle more permissive, and perhaps conducive, to multipartitism in plants and monopartite segmentation in insects.

Finally, a recent study on RVFV showed that complementation between viral particles containing incomplete genomes occurs beyond the within-cell level^41^. This study showed that coinfecting viral particles with incomplete genomes could complement each other and be successfully acquired by vectors in which they can produce viral particles containing genomes. Thus, particles with incomplete genomes may significantly contribute to the between-host transmission of these monopartite segmented viruses reminiscent of the non-concomitant transmission observed in the multipartite FBNSV^8^.

## Monopartite nonsegmented viruses

The sections above discuss several features associated with the multipartite architecture of viral genomes and how these may be extended to at least some monopartite segmented viruses. While much more speculative at this stage, we wish to briefly mention potential analogies in monopartite nonsegmented viruses, in particular with respect to the possibility of gene expression regulation through gene copy number differential modification, and the mechanisms facilitating the maintenance of genomic integrity.

The question of the GF and gene copy number variation is an interesting prospect for monopartite nonsegmented viruses. Gene copy number variation has thus far been considered and studied solely through gene amplification and gene accordion processes^45,46^. In this case, a gene can be duplicated several times, up to tens of copies, within a monopartite nonsegmented genome, inducing the adaptive overexpression of the amplified gene and the selection of the corresponding genome variant. This process appears possible only in large dsDNA viruses, which have viral particles that can accommodate important genome length variations. Logically, gene copy number variation through gene amplification is considered impossible in small ssDNA or RNA monopartite nonsegmented viruses where the genome length is highly constrained within small icosahedral particles^46^. However, the production and accumulation of myriads of diverse defective genomes (DGs) in all virus infections^47^, each DG type containing only a subset of the viral genes, could result in variation of gene copy numbers and so variation in gene expression. To our knowledge, a situation where the production and regulation of the DG population would engender a phenomenon analogous to the GF in monopartite nonsegmented viruses has never been formally investigated. Nevertheless, it has been argued that DG-related variation of gene copy number may, at least in some cases, provide advantages to the viruses^26,48–50^. It is thus possible to imagine that even some monopartite nonsegmented viruses could generate a GF during cell and host infection. In this speculation, a possible though not exclusive mechanism allowing the maintenance of genome integrity could be the preferential packaging and transmission of non-defective full-length genomes linked to genome length constraints. In any case, if it were true that monopartite nonsegmented viruses can as well generate GFs through the extemporaneous production of DGs, this would indicate that any potential advantage incurred by GFs is not the driving force leading to genome segmentation.

In addition to DGs that are generated from the viral genome, monopartite nonsegmented viruses are sometimes associated with satellites^51^. Both satellites and DGs can be beneficial^47^, and they need complementation by the helper virus. The maintenance of these successful associations may profit from analogous mechanisms as those revealed for FBNSV: the within hosts multicellular way of life, and the non-concomitant between-hosts transmission of helper virus and satellite. While there is no theoretical hindrance to these mechanisms, none have been explicitly envisaged and investigated thus far in a monopartite nonsegmented viral system.

Altogether, the studies discussed above suggest that the frontier between multipartite, monopartite segmented, and perhaps even monopartite nonsegmented viruses may be much more flexible than anticipated. Even though the specific manifestations of phenomena such as GFs, non-concomitant gene transmission, and multicellular way of life, may depend on the viral packaging strategies, their essence in allowing flexible and rapidly modulable gene expression and complementation beyond the single host cell level could be much more shared than previously thought. This possibility opens avenues to future research, making it perhaps necessary to reconsider the extent of multicomponent viral systems in the virus world on how they function and evolve.

## References

[1] Michalakis, Y. & Blanc, S. The curious strategy of multipartite viruses. *Annu. Rev. Virol.* **7**, 203–218 (2020). 32991271 10.1146/annurev-virology-010220-063346

[2] Sicard, A., Michalakis, Y., Gutiérrez, S. & Blanc, S. The strange lifestyle of multipartite viruses. *PLoS Pathog.* **12**, e1005819 (2016). 27812219 10.1371/journal.ppat.1005819 PMC5094692

[3] Sicard, A. et al. Gene copy number is differentially regulated in a multipartite virus. *Nat. Commun.* **4**, 2248 (2013). 23912259 10.1038/ncomms3248

[4] Gallet, R. et al. Gene copy number variations at the within-host population level modulate gene expression in a multipartite virus. *Virus Evol.* **8**, veac058 (2022). 35799884 10.1093/ve/veac058 PMC9255600

[5] Sicard, A. et al. Circulative nonpropagative aphid transmission of nanoviruses: an oversimplified view. *J. Virol.* **89**, 9719–9726 (2015). 26178991 10.1128/JVI.00780-15 PMC4577921

[6] Zwart, M. P. & Elena, S. F. Modeling multipartite virus evolution: the genome formula facilitates rapid adaptation to heterogeneous environments†. *Virus Evol.* **6**, veaa022 (2020). 32405432 10.1093/ve/veaa022 PMC7206449

[7] Sicard, A. et al. A multicellular way of life for a multipartite virus. *eLife* **8**, e43599 (2019). 30857590 10.7554/eLife.43599 PMC6414197

[8] Di Mattia, J. et al. Nonconcomitant host-to-host transmission of multipartite virus genome segments may lead to complete genome reconstitution. *Proc. Natl. Acad. Sci. USA* **119**, e2201453119 (2022). 35914138 10.1073/pnas.2201453119 PMC9371732

[9] Michalakis, Y. & Blanc, S. Editorial overview: multicomponent viral systems. *Curr. Opin. Virol.* **33**, vi–ix (2018). 30527269 10.1016/j.coviro.2018.11.009

[10] Lucía-Sanz, A. & Manrubia, S. Multipartite viruses: adaptive trick or evolutionary treat? *Npj Syst. Biol. Appl.* **3**, 34 (2017). 29263796 10.1038/s41540-017-0035-y PMC5680193

[11] Xiao, Y.-X., Li, D., Wu, Y.-J., Liu, S.-S. & Pan, L.-L. Constant ratio between the genomic components of bipartite begomoviruses during infection and transmission. *Virol. J.* **20**, 1–10 (2023). 37605144 10.1186/s12985-023-02148-2 PMC10464424

[12] Kennedy, G. G. et al. Genome segment ratios change during whitefly transmission of two bipartite cassava mosaic begomoviruses. *Sci. Rep.* **13**, 10059 (2023). 37344614 10.1038/s41598-023-37278-8 PMC10284885

[13] Hu, Z. et al. Genome segments accumulate with different frequencies in *Bombyx mori* bidensovirus. *J. Basic Microbiol.* **56**, 1338–1343 (2016). 27160646 10.1002/jobm.201600120

[14] Sánchez-Navarro, J. A., Zwart, M. P. & Elena, S. F. Effects of the number of genome segments on primary and systemic infections with a multipartite plant RNA virus. *J. Virol.* **87**, 10805–10815 (2013). 23903837 10.1128/JVI.01402-13 PMC3807391

[15] Wu, B., Zwart, M. P., Sánchez-Navarro, J. A. & Elena, S. F. Within-host evolution of segments ratio for the tripartite genome of Alfalfa Mosaic virus. *Sci. Rep.* **7**, 5004 (2017). 28694514 10.1038/s41598-017-05335-8 PMC5504059

[16] Feng, J.-L. et al. Quantitative determination of *cucumber mosaic virus* genome RNAs in virions by real-time reverse transcription-polymerase chain reaction. *Acta Biochim. Biophys. Sin.* **38**, 669–676 (2006). 17033712 10.1111/j.1745-7270.2006.00216.x

[17] Boezen, D., Johnson, M. L., Grum-Grzhimaylo, A. A., van der Vlugt, R. A. & Zwart, M. P. Evaluation of sequencing and PCR-based methods for the quantification of the viral genome formula. *Virus Res* **326**, 199064 (2023). 36746340 10.1016/j.virusres.2023.199064 PMC10194290

[18] Boezen, D. et al. Mixed viral infection constrains the genome formula of multipartite cucumber mosaic virus. *Front. Virol.* **3**, 1225818 (2023).

[19] Bashir, S. et al. Quantitation of multipartite banana bunchy top virus genomic components and their transcripts in infected tissues of banana (*Musa acuminata*). *Agronomy* **12**, 2990 (2022).

[20] Guyot, V. et al. A newly emerging alphasatellite affects banana bunchy top virus replication, transcription, siRNA production and transmission by aphids. *PLoS Pathog* **18**, e1010448 (2022). 35413079 10.1371/journal.ppat.1010448 PMC9049520

[21] Yu, N.-T. et al. Independent modulation of individual genomic component transcription and a cis-acting element related to high transcriptional activity in a multipartite DNA virus. *BMC Genom.* **20**, 573 (2019). 10.1186/s12864-019-5901-0 PMC6625112 31296162

[22] Bonnamy, M., Brousse, A., Pirolles, E., Michalakis, Y. & Blanc, S. The genome formula of a multipartite virus is regulated both at the individual segment and the segment group levels. *PLoS Pathog.* **20**, e1011973 (2024). 38271470 10.1371/journal.ppat.1011973 PMC10846721

[23] Noda, T. et al. Architecture of ribonucleoprotein complexes in influenza A virus particles. *Nature* **439**, 490–492 (2006). 16437116 10.1038/nature04378

[24] Hutchinson, E. C., von Kirchbach, J. C., Gog, J. R. & Digard, P. Genome packaging in influenza A virus. *J. Gen. Virol.* **91**, 313–328 (2010). 19955561 10.1099/vir.0.017608-0

[25] Brooke, C. B. et al. Most Influenza A virions fail to express at least one essential viral protein. *J. Virol.* **87**, 3155–3162 (2013). 23283949 10.1128/JVI.02284-12 PMC3592173

[26] Diefenbacher, M., Sun, J. & Brooke, C. B. The parts are greater than the whole: the role of semi-infectious particles in influenza A virus biology. *Curr. Opin. Virol.* **33**, 42–46 (2018). 30053722 10.1016/j.coviro.2018.07.002 PMC6642613

[27] Nakatsu, S. et al. Complete and incomplete genome packaging of influenza A and B viruses. *mBio* **7**, e01248–16 (2016). 27601575 10.1128/mBio.01248-16 PMC5013298

[28] Jacobs, N. T. et al. Incomplete influenza A virus genomes occur frequently but are readily complemented during localized viral spread. *Nat. Commun.* **10**, 1–17 (2019). 31387995 10.1038/s41467-019-11428-x PMC6684657

[29] Farrell, A., Phan, T., Brooke, C. B., Koelle, K. & Ke, R. Semi-infectious particles contribute substantially to influenza virus within-host dynamics when infection is dominated by spatial structure. *Virus Evol.* **9**, vead020 (2023). 37538918 10.1093/ve/vead020 PMC10395763

[30] Martin, B. E., Harris, J. D., Sun, J., Koelle, K. & Brooke, C. B. Cellular co-infection can modulate the efficiency of influenza A virus production and shape the interferon response. *PLoS Pathog.* **16**, e1008974 (2020). 33064776 10.1371/journal.ppat.1008974 PMC7592918

[31] Brooke, C. B., Ince, W. L., Wei, J., Bennink, J. R. & Yewdell, J. W. Influenza A virus nucleoprotein selectively decreases neuraminidase gene-segment packaging while enhancing viral fitness and transmissibility. *Proc. Natl. Acad. Sci. USA* **111**, 16854–16859 (2014). 25385602 10.1073/pnas.1415396111 PMC4250133

[32] Roberts, K. L., Manicassamy, B. & Lamb, R. A. Influenza A virus uses intercellular connections to spread to neighboring cells. *J. Virol.* **89**, 1537–1549 (2015). 25428869 10.1128/JVI.03306-14 PMC4300760

[33] Kumar, A. et al. Influenza virus exploits tunneling nanotubes for cell-to-cell spread. *Sci. Rep.* **7**, 40360 (2017). 28059146 10.1038/srep40360 PMC5216422

[34] Ganti, K., Han, J., Manicassamy, B. & Lowen, A. C. Rab11a mediates cell-cell spread and reassortment of influenza A virus genomes via tunneling nanotubes. *PLoS Pathog.* **17**, e1009321 (2021). 34473799 10.1371/journal.ppat.1009321 PMC8443049

[35] Borodavka, A., Desselberger, U. & Patton, J. T. Genome packaging in multi-segmented dsRNA viruses: distinct mechanisms with similar outcomes. *Curr. Opin. Virol.* **33**, 106–112 (2018). 30145433 10.1016/j.coviro.2018.08.001 PMC6289821

[36] Moreau, Y. et al. The genome segments of bluetongue virus differ in copy number in a host-specific manner. *J. Virol.* **95**, e01834–20 (2020). 33028716 10.1128/JVI.01834-20 PMC7737730

[37] Wichgers Schreur, P. J. & Kortekaas, J. Single-molecule FISH reveals non-selective packaging of Rift Valley fever virus genome segments. *PLoS Pathog* **12**, e1005800 (2016). 27548280 10.1371/journal.ppat.1005800 PMC4993503

[38] Wichgers Schreur, P. J., Kormelink, R. & Kortekaas, J. Genome packaging of the Bunyavirales. *Curr. Opin. Virol.* **33**, 151–155 (2018). 30227361 10.1016/j.coviro.2018.08.011

[39] Zhao, W., Wang, Q., Xu, Z., Liu, R. & Cui, F. Distinct replication and gene expression strategies of the Rice Stripe virus in vector insects and host plants. *J. Gen. Virol.* **100**, 877–888 (2019). 30990404 10.1099/jgv.0.001255

[40] Yvon, M. et al. The genome of a bunyavirus cannot be defined at the level of the viral particle but only at the scale of the viral population. *Proc. Natl. Acad. Sci. USA* **120**, e2309412120 (2023). 37983500 10.1073/pnas.2309412120 PMC10691328

[41] Bermúdez-Méndez, E. et al. Incomplete bunyavirus particles can cooperatively support virus infection and spread. *PLoS Biol.* **20**, e3001870 (2022). 36378688 10.1371/journal.pbio.3001870 PMC9665397

[42] Bermúdez-Méndez, E., Katrukha, E. A., Spruit, C. M., Kortekaas, J. & Wichgers Schreur, P. J. Visualizing the ribonucleoprotein content of single bunyavirus virions reveals more efficient genome packaging in the arthropod host. *Commun. Biol.* **4**, 1–13 (2021). 33753850 10.1038/s42003-021-01821-y PMC7985392

[43] de Oliveira Resende, R. et al. Generation of envelope and defective interfering RNA mutants of tomato spotted wilt virus by mechanical passage. *J. Gen. Virol.* **72**, 2375–2383 (1991). 1919523 10.1099/0022-1317-72-10-2375

[44] Nagata, T., Inoue-Nagata, A. K., Prins, M., Goldbach, R. & Peters, D. Impeded thrips transmission of defective *Tomato spotted wilt virus* isolates. *Phytopathology* **90**, 454–459 (2000). 18944549 10.1094/PHYTO.2000.90.5.454

[45] Elde, N. C. et al. Poxviruses deploy genomic accordions to adapt rapidly against host antiviral defenses. *Cell* **150**, 831–841 (2012). 22901812 10.1016/j.cell.2012.05.049 PMC3499626

[46] Bayer, A., Brennan, G. & Geballe, A. P. Adaptation by copy number variation in monopartite viruses. *Curr. Opin. Virol.* **33**, 7–12 (2018). 30015083 10.1016/j.coviro.2018.07.001 PMC6289852

[47] Vignuzzi, M. & López, C. B. Defective viral genomes are key drivers of the virus–host interaction. *Nat. Microbiol.* **4**, 1075 (2019). 31160826 10.1038/s41564-019-0465-y PMC7097797

[48] Gutiérrez, S. & Zwart, M. P. Population bottlenecks in multicomponent viruses: first forays into the uncharted territory of genome-formula drift. *Curr. Opin. Virol.* **33**, 184–190 (2018). 30471620 10.1016/j.coviro.2018.09.001

[49] Bull, J. C., Godfray, H. C. J. & O’Reilly, D. R. Persistence of an occlusion-negative recombinant nucleopolyhedrovirus in *Trichoplusia ni* indicates high multiplicity of cellular infection. *Appl. Environ. Microbiol.* **67**, 5204–5209 (2001). 11679346 10.1128/AEM.67.11.5204-5209.2001 PMC93291

[50] Bull, J. C., Godfray, H. C. J. & O’Reilly, D. R. A few-polyhedra mutant and wild-type nucleopolyhedrovirus remain as a stable polymorphism during serial coinfection in *Trichoplusia ni*. *Appl. Environ. Microbiol.* **69**, 2052–2057 (2003). 12676682 10.1128/AEM.69.4.2052-2057.2003 PMC154768

[51] Gnanasekaran, P. & Chakraborty, S. Biology of viral satellites and their role in pathogenesis. *Curr. Opin. Virol.* **33**, 96–105 (2018). 30144641 10.1016/j.coviro.2018.08.002

[52] Mansourpour, M. et al. Effects of an alphasatellite on the life cycle of the nanovirus *Faba bean necrotic yellows virus*. *J. Virol.* **96**, e01388-21 (2022). 34818072 10.1128/JVI.01388-21 PMC8826813

[53] Feng, J., Zeng, R. & Chen, J. Accurate and efficient data processing for quantitative real-time PCR using a tripartite plant virus as a model. *BioTechniques* **44**, 901–912 (2008). 18533900 10.2144/000112750

[54] Dall’Ara, M., Guo, Y., Poli, D., Gilmer, D. & Ratti, C. Analysis of the relative frequencies of the multipartite BNYVV genomic RNAs in different plants and tissues. *J. Gen. Virol.* **105**, 001950 (2024). 10.1099/jgv.0.001950 38197877

